# The prognostic and predictive value of *ESR1* fusion gene transcripts in primary breast cancer

**DOI:** 10.1186/s12885-022-09265-1

**Published:** 2022-02-12

**Authors:** Silvia R. Vitale, Kirsten Ruigrok-Ritstier, A. Mieke Timmermans, Renée Foekens, Anita M. A. C. Trapman-Jansen, Corine M. Beaufort, Paolo Vigneri, Stefan Sleijfer, John W. M. Martens, Anieta M. Sieuwerts, Maurice P. H. M. Jansen

**Affiliations:** 1 Department of Medical Oncology, Erasmus MC Cancer Institute, Erasmus University Medical Center, Rotterdam, The Netherlands; 2 Department of Clinical and Experimental Medicine, University of Catania, 95123 Catania, Italy; 3 Center of Experimental Oncology and Hematology, A.O.U. Policlinico “G. Rodolico- San Marco”, 95123 Catania, Italy; 4 Cancer Genomics Netherlands, Rotterdam, The Netherlands

**Keywords:** Fusion genes, *ESR1*, *CCDC170*, Breast cancer, Prognosis, RT-qPCR

## Abstract

**Background:**

In breast cancer (BC), recurrent fusion genes of estrogen receptor alpha (*ESR1*) and *AKAP12*, *ARMT1* and *CCDC170* have been reported. In these gene fusions the ligand binding domain of ESR1 has been replaced by the transactivation domain of the fusion partner constitutively activating the receptor. As a result, these gene fusions can drive tumor growth hormone independently as been shown in preclinical models, but the clinical value of these fusions have not been reported. Here, we studied the prognostic and predictive value of different frequently reported *ESR1* fusion transcripts in primary BC.

**Methods:**

We evaluated 732 patients with primary BC (131 *ESR1-*negative and 601 *ESR1-*positive cases), including two ER-positive BC patient cohorts: one cohort of 322 patients with advanced disease who received first-line endocrine therapy (ET) (predictive cohort), and a second cohort of 279 patients with lymph node negative disease (LNN) who received no adjuvant systemic treatment (prognostic cohort). Fusion gene transcript levels were measured by reverse transcriptase quantitative PCR. The presence of the different fusion transcripts was associated, in uni- and multivariable Cox regression analysis taking along current clinico-pathological characteristics, to progression free survival (PFS) during first-line endocrine therapy in the predictive cohort, and disease- free survival (DFS) and overall survival (OS) in the prognostic cohort.

**Results:**

The *ESR1-CCDC170* fusion transcript was present in 27.6% of the *ESR1*-positive BC subjects and in 2.3% of the *ESR1*-negative cases. In the predictive cohort, none of the fusion transcripts were associated with response to first-line ET. In the prognostic cohort, the median DFS and OS were respectively 37 and 93 months for patients with an *ESR1-CCDC170* exon 8 gene fusion transcript and respectively 91 and 212 months for patients without this fusion transcript. In a multivariable analysis, this *ESR1*-*CCDC170* fusion transcript was an independent prognostic factor for DFS (HR) (95% confidence interval (CI): 1.8 (1.2–2.8), *P* = 0.005) and OS (HR (95% CI: 1.7 (1.1–2.7), *P* = 0.023).

**Conclusions:**

Our study shows that in primary BC only *ESR1-CCDC170* exon 8 gene fusion transcript carries prognostic value. None of the *ESR1* fusion transcripts, which are considered to have constitutive ER activity, was predictive for outcome in BC with advanced disease treated with endocrine treatment.

**Supplementary Information:**

The online version contains supplementary material available at 10.1186/s12885-022-09265-1.

## Background

The estrogen receptor (ER) plays a key role in cellular growth and tumor development in a large fraction of breast cancers. As a result, endocrine therapy has been and still is a successful treatment in patients with *ESR1*-positive (*ESR1* +) breast cancers (BC) [1]. However, in the metastatic setting, nearly half of the patients are de novo resistant to endocrine therapy while the remaining cases acquire resistance over time [2, 3]. One of the primary characterized mechanisms of acquired resistance to endocrine therapy is the acquisition of mutations within the ligand-binding domain (LBD) of the estrogen receptor alpha gene (*ESR1*) activating the receptor constitutively thereby rendering tumor cells less dependent on estrogen [4–7]. Another mechanism that lead to less estrogen dependency of BC cells is the occurrence of ESR1 fusion proteins. Through analysis of RNA-sequencing data in breast cancer, recurrent intragenic fusions of 5′ end of *ESR1* and the 3′ ends of *AKAP12*, *ARMT1* or *CCDC170* amongst other genes have been identified [8–13]. *AKAP12*, *ARMT1, and CCDC170* genes together with *ESR1* gene were selected for our evaluation, because they all were located at the 6q25.1 locus within 1 Mb distance [14] and fusions between the two non-coding 5’ exons of *ESR1* with the 3’ ends of *CCDC170*, *AKAP12* and *ARMT*1, upstream of *ESR1*, were identified in patients resistant to endocrine treatment [9, 10].

Gene fusions were preferentially detected in high-grade disease and/or endocrine-resistant forms of *ESR1* + BC [10, 13]. Particularly, an enrichment of *ESR1-CCDC170* fusion was previously reported in HER-positive patients (luminal A 9%, luminal B 3–8% and HER2 3.1%) and was correlated with a worse clinical outcome after endocrine therapy [9, 15, 16]. The *ESR1-AKAP12* fusion was identified in 6.5% breast cancer that were resistant to letrozole aromatase inhibitor treatment [17].The novel fusion *ESR1-ARMT1* was instead detected in a HER2-negative patient with luminal A-like subtype [16] and in a breast cancer patient who had not received endocrine therapy [18]. Moreover, a recently study based on molecular characterization of luminal breast cancer in African American women reported the fusions at a frequency of 11% for *ESR1-CCDC170,* 8% for *ESR1-AKAP12* and 6% for *ESR1-ARMT1 *[19]. Despite the diversity among these fusions, they share a common structure retaining the hormone-independent transactivation domain as well as the DNA-binding domain whereas their ligand-binding domain is lost and replaced with a functional (transactivating) domain of the fusion partner, suggesting a pathological impact in *ESR1* + BC [13]. However, the clinical significance of these fusions has not yet been properly addressed in uniform and well annotated cohorts.

In this study, we explored the occurrence of fusion transcripts of three of the most commonly reported fusion partners of *ESR1* (i.e. *CCDC170*, *AKAP12* and *ARMT1*) and determined the associations of their presence with clinical outcome in a cohort of 732 breast cancer patients allowing us to investigate their predictive value for endocrine treatment failure as well as their prognostic value.

## Methods

### Study cohorts

The protocol to study biological markers associated with disease outcome was approved by the medical ethics committee of the Erasmus Medical Centre Rotterdam, The Netherlands (MEC 02.953) and was performed in accordance with the Code of Conduct of the Federation of Medical Scientific Societies in The Netherlands (https://www.federa.org/codes-conduct). The use of coded left-over material for scientific purposes and, therefore, for the greater good, does not require informed consent according to Dutch law and the new European general data protection regulation (GDPR).

**Fig. 1:**
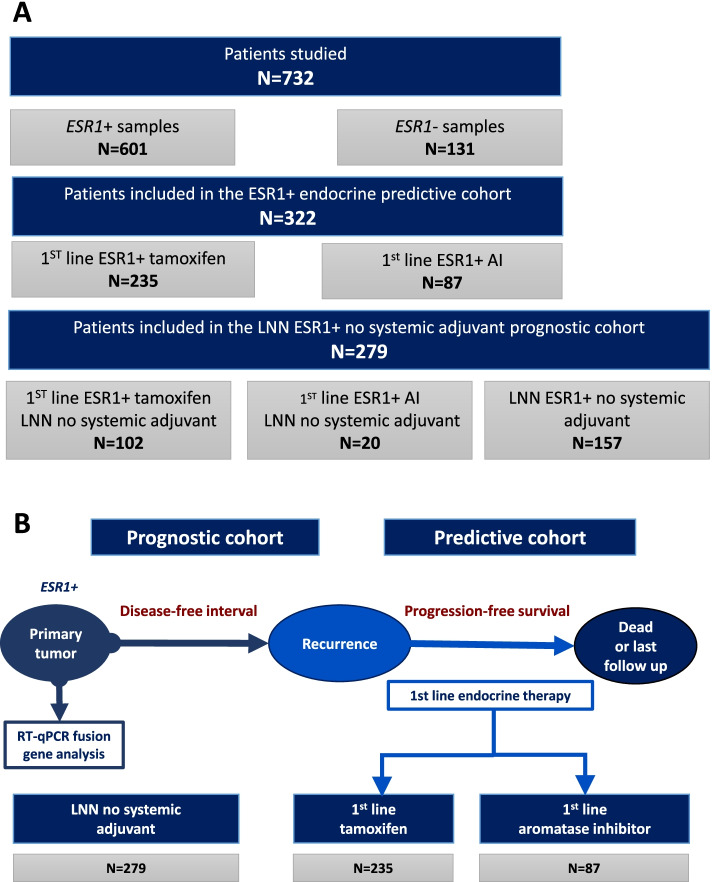
Overview of the study and selection of available patients. **A** Flow diagram of the study; **B** Workflow of processing samples: fusion gene mRNA levels were measured in 322 ER-positive primary tumors (predictive cohort) by quantitative reverse transcriptase PCR (RT-qPCR). All patients in this cohort were hormone-naïve and all experienced a disease recurrence and subsequently received 1^st^ line endocrine therapy. The association of the presence of *ESR1* fusion genes in the primary tumor progression-free survival (PFS) after start with 1^st^ line tamoxifen (*n* = 235) or aromatase inhibitors (*n* = 87), were evaluated. Similarly, disease free interval (DFS) and overall survival (OS) were investigated in 279 lymph node negative ER-positive breast cancer patients (prognostic cohort) who had not received any (neo)adjuvant systemic therapy. *ESR1*: Estrogen Receptor 1 gene; AI: Aromatase Inhibitor; LNN: Lymph node negative; ER: Estrogen Receptor; RT-qPCR: Quantitative reverse transcriptase PCR

In this retrospective study (see Fig. 1A for the consort diagram of the study), female patients were included, who underwent surgery for invasive primary breast cancer between 1980 and 2000 in the Netherlands. A further selection criterion was no previously diagnosed cancers with the exception of basal cell carcinoma or stage Ia/Ib cervical cancer. Within this study, only data from sections of primary tumors with at least 30% invasive tumor cells were included. The details of tissue processing, RNA isolation, cDNA synthesis and QC of this cohort have been described previously [20, 21]. Tumor grade was assessed according to standard procedures at the time of inclusion. For the classification of patients’ RNA samples regarding expression of the estrogen and progesterone receptors, as well as the human epidermal growth factor receptor 2 (HER2) amplification status, reverse transcriptase quantitative PCR (RT-qPCR) was used with cut-offs previously described by us [20, 21].

The total cohort consisted of 732 patients with primary breast cancer (131 *ESR1*-negative and 601 *ESR1-*positive cases) (Fig. 1B). The clinical relevance of the gene fusion transcripts was evaluated in a predictive and a prognostic cohort of *ESR1* + BC patients.

The predictive cohort consisted of 322 breast cancer patients with *ESR1* + primary tumors of which 235 patients received tamoxifen (40 mg daily) and 87 patients an aromatase inhibitor (AI: anastrozole, letrozole, exemestane [22]) as a 1^st^-line treatment for recurrent disease. Clinical response to tamoxifen therapy was defined as previously described [20, 23]. The prognostic cohort included primary tumors from 279 lymph node negative (LNN) *ESR1* + BC patients who had not received any systemic (neo) adjuvant therapy. Of note, 122 of these LNN *ESR1* + patients were also included in the predictive cohort. Clinicopathological characteristics of each of these 2 cohorts are described in Table 1 Association of *ESR1* fusions with clinical parameters of patients enrolled in the predictive cohort and in the prognostic cohort are reported in Table 2 and Table 3, respectively.

**Table 1:** Clinicopathological characteristics of ER-positive breast cancer patient cohorts

	**Predictive Endocrine Therapy Cohorts**		**Prognostic Cohort**
	**Tamoxifen**	**Aromatase inhibitors**	**Lymph node negative (LNN)**
**Total**	235	87	279
**Median age (range)**	61 (29–90)	66 (35–86)	55 (26–85)
**Menopausal Status:**			
Premenopausal	60	4	120
Postmenopausal	175	82	159
**Surgery:**			
Lumpectomy	87	8	178
Ablation	147	22	101
**Adjuvant hormonal therapy:**			
no	235	17	279
yes	0	69	0
**Adjuvant chemotherapy:**			
no	198	69	279
yes	37	18	0
**Lymph node status:**			
negative	102	20	279
positive	81	49	0
not applicable (M1)	42	17	0
**Distant metastasis:**			
yes	235	87	165
no	0	0	114
**Disease -Free Interval:**			
< 1 year	59	13	20
1–3 year	108	29	71
> 3 year	68	45	188
**Median Follow-up time (in months):**			
after surgery	62 (3–272)	103 (7–295)	93 (5–337)
after start therapy	30 (1–208)	45 (2–108)	
**PR status**^**a**^**:**			
Positive	186	72	217
Negative	48	15	62
**HER2 status**^**a**^**:**			
Amplified	31	10	43
Not amplified	202	77	233
**CCDC170 status**^**a**^**:**			
Positive	206	81	252
Negative	28	3	26

*ESR1* estrogen receptor alpha, *LNN* lymph node negative disease, *M1* methastatic stage 1, *PR* progesterone receptor, *HER2* human epidermal growth factor receptor 2, *CCDC170* coiled-coil domain containing 170, *RT-qPCR* Quantitative Real-Time Polymerase Chain Reaction

^a^as measured by RT-qPCR

**Table 2:** Association of *ESR1* fusions with clinical parameters in the predictive cohort

		**Predictive Endocrine Therapy Cohorts**											
**Parameters**	**n**	***at least one ESR1-CCDC170*** ***(exon 2 to 8) fusion***		***P-Value***	***ESR1-CCDC170 (exon 2) fusion***		***PValue***	***ESR1-CCDC170 (exon 8) fusion***		***P-Value***	***ESR1-AKAP12***		***P-Value***
		**n**	**%**		**n**	**%**		**n**	**%**		***n***	***%***	
**All patients**	322	89	27.6%		50	15.5%		51	15.8%		13	4.0%	
**Age at start 1**^**st**^** line treatment (years)**													
≤ 50	63	19	30.2%	*0.63*	12	19.0%	*0.62*	8	12.7%	***0.029***	1	1.6%	*0.36*
> 50- ≤ 70	161	37	23.0%		23	14.3%		24	14.9%		7	4.3%	
> 70	98	33	33.7%		15	15.3%		19	19.4%		5	5.1%	
**Menopausal status at start of 1**^**st**^** line treatment**													
Premenopausal	64	17	26.6%	*0.82*	10	15.6%	*0.99*	8	12.5%	*0.41*	1	1.6%	*0.26*
Postmenopausal	257	72	28.0%		40	15.6%		43	16.7%		12	4.7%	
**Surgery type**													
Lumpectomy	95	25	26.3%	*0.79*	14	14.7%	*0.90*	15	15.8%	*0.83*	2	2.1%	*0.89*
Ablation	169	42	24.9%		24	14.2%		25	14.8%		4	2.4%	
**Radiotherapy**													
No	105	30	28.6%	*0.33*	20	19.0%	*0.08*	16	15.2%	*0.98*	2	1.9%	*0.74*
Yes	159	37	23.3%		18	11.3%		24	15.1%		4	2.5%	
**Nodal status**													
No lymph nodes	122	33	27.0%	*0.88*	19	15.6%	*0.99*	20	16.4%	*0.95*	4	3.3%	*0.2*
Positive lymph nodes	130	38	29.2%		21	16.2%		22	16.9%		9	6.9%	
Tumor outside lymph nodes	53	15	28.3%		8	15.1%		7	13.2%		0	0.0%	
Not applicable (M1)	16	3	18.8%		2	12.5%		2	12.5%		0	0.0%	
**Pathological Tumor classification**													
pT1	85	22	25.9%	*0.60*	13	15.3%	*0.21*	14	16.5%	*0.90*	2	2.4%	*0.36*
pT2 + unknown	186	50	26.9%		25	13.4%		30	16.1%		10	5.4%	
pT3 + pT4	51	17	33.3%		12	23.5%		7	13.7%		1	2.0%	
**Tumor grade**													
Poor	160	45	28.1%	*0.36*	27	16.9%	*0.60*	27	16.9%	*0.60*	7	4.4%	*0.078*
Unknown	81	18	22.2%		10	12.3%		10	12.3%		0	0.0%	
Moderate/Good	74	24	32.4%		13	17.6%		13	17.6%		5	6.8%	
**Tumor cell content**													
30–49%	27	7	25.9%	*0.96*	4	14.8%	*0.99*	2	7.4%	*0.25*	2	7.4%	*0.63*
50–70%	98	28	28.6%		15	15.3%		13	13.3%		4	4.1%	
> 70%	197	54	27.4%		31	15.7%		36	18.3%		7	3.6%	
**Hormone/ growth factor status (RT-qPCR)**													
*ESR1-*negative	0	0			0			0			0		
*ESR1-*positive	322	89	27.6%		50	15.5%		51	15.8%		13	4.0%	
*PR-*negative	63	18	28.6%	*0.87*	11	17.5%	*0.65*	11	17.5%	*0.70*	6	9.5%	***0.014***
*PR-*positive	258	71	27.5%		39	15.1%		40	15.5%		7	2.7%	
*HER2* non-amplified	279	77	27.6%	*0.63*	44	15.8%	*0.85*	45	16.1%	*0.81*	13	4.7%	*0.16*
*HER2* amplified	41	12	29.3%		6	14.6%		6	14.6%		0	0.0%	
*CCDC170* negative	31	5	16.1%	*0.13*	2	6.5%	*0.15*	4	12.9%	*0.62*	0	0.0%	*0.23*
*CCDC170* positive	287	83	28.9%		47	16.4%		47	16.4%		13	4.5%	
**Adjuvant endocrine therapy**													
No	252	66	26.2%	*0.24*	38	15.1%	*0.64*	36	14.3%	*0.13*	7	2.8%	***0.030***
Yes (AI cohort only)	69	23	33.3%		12	17.4%		15	21.7%		6	8.7%	
**Adjuvant chemotherapy**													
No	267	76	28.5%	*0.47*	40	15.0%	*0.55*	45	16.9%	*0.27*	12	4.5%	*0.36*
Yes	55	13	23.6%		10	18.2%		6	10.9%		1	1.8%	
**Disease-free interval**													
≤ 1 year disease-free	72	23	31.9%	*0.47*	14	19.4%	*0.62*	12	16.7%	*0.99*	2	2.8%	*0.45*
1–3 years disease-free	137	37	27.0%		20	14.6%		20	14.6%		8	5.8%	
> 3 years disease-free	113	29	25.7%		16	14.2%		19	16.8%		3	2.7%	
**Dominant site of metastasis**													
Local regional	29	10	34.5%	*0.51*	7	24.1%	*0.32*	4	13.8%	*0.36*	0	0.0%	*0.40*
Bone	159	40	25.2%		25	15.7%		21	13.2%		6	3.8%	
Other distant metastasis	130	38	29.2%		17	13.1%		25	19.2%		7	5.4%	
**Response type**													
Complete response	11	3	27.3%	*0.87*	2	18.2%	*0.73*	1	9.1%	*0.29*	0	0.0%	*0.46*
Partial response	39	9	23.1%		3	7.7%		6	15.4%		2	5.1%	
Stable disease over 6 months (SD > 6 m)	115	32	27.8%		16	13.9%		23	20.0%		1	0.9%	
Stable disease for 6 months or less (SD ≤ 6 m)	13	2	15.4%		2	15.4%		1	7.7%		0	0.0%	
Progressive disease (PD)	83	20	24.1%		14	16.9%		8	9.6%		3	3.6%	
**Response type**													
No response	96	22	22.9%	*0.50*	16	16.7%	*0.38*	9	9.4%	*0.05*	3	3.1%	*0.50*
Response	165	44	26.7%		21	12.7%		30	18.2%		3	1.8%	

*ESR1* estrogen receptor alpha, *CCDC170* coiled-coil domain containing 170, *AKAP12* A-Kinase Anchoring Protein 12 gene, *ESR1-CCDC170* ESR1-CCDC170 gene fusion, *ESR1-AKAP12* ESR1-AKAP12 gene fusion, *M1* methastatic stage 1, *pT* primary tumor, *pT1* small primary tumor (tumour is 2 cm across or less), *pT2* tumour more than 2 cm but no more than 5 cm across, *pT3* T3 tumour bigger than 5 cm across, *pT4* tumor with phatological stage, *RT-qPCR* Quantitative Real-Time Polymerase Chain Reaction, *PR* progesterone receptor, *HER2* human epidermal growth factor receptor, *AI* aromatase inhibitors, *SD* standard deviation, *PD* progressive disease

Statistically significant differences are indicated in bold

**Table 3:** Associations of *ESR1* fusions with clinical parameters in prognostic clinical cohort

		**LNN ESR + Prognostic cohort**												
**Parameters**		**n**	***at least one ESR1-CCDC170*** ***(exon 2 to 8) fusion***		***P-value***	***ESR1-CCDC170 (exon 2) fusion***		***P-value***	***ESR1-CCDC170 (exon 8) fusion***		***P-value***	***ESR1-AKAP12***		***P-value***
			**n**	**%**		**n**	**%**		**n**	**%**		***n***	***%***	
**All patients**		279	70	25.1%		33	11.8%		39	14.0%		5	1.8%	
**Age at primary surgery**														
≤ 40 years		29	6	20.7%	***0.001***	4	13.8%	*0.38*	4	13.8%	*0.26*	1	3.4%	*0.27*
41–50 years		81	11	13.6%		5	6.2%		5	6.2%		0	0.0%	
51–70 years		125	36	28.8%		16	12.8%		21	16.8%		3	2.4%	
> 70 years		44	17	38.6%		8	18.2%		9	20.5%		1	2.3%	
**Menopausal status**														
Premenopausal		120	19	15.8%	***0.002***	10	8.3%	*0.12*	11	9.2%	***0.044***	1	0.8%	*0.29*
Postmenopausal		159	51	32.1%		23	14.5%		28	17.6%		4	2.5%	
**Surgery type**														
Lumpectomy		178	44	24.7%	*0.85*	19	10.7%	*0.43*	25	14.0%	*0.97*	4	2.2%	*0.45*
Ablation		101	26	25.7%		14	13.9%		14	13.9%		1	1.0%	
**Radiotherapy**														
No		84	24	28.6%	*0.38*	14	16.7%	*0.10*	12	14.3%	*0.92*	1	1.2%	*0.62*
Yes		195	46	23.6%		19	9.7%		27	13.8%		4	2.1%	
**Nodal status**														
No lymph nodes		279	70	25.1%		33	11.8%		39	14.0%		5	1.8%	
Positive lymph nodes		0	0			0			0			0		
Tumor outside lymph nodes		0	0			0			0			0		
**Pathological Tumor classification**														
pT1		151	34	22.5%	*0.28*	17	11.3%	*0.61*	16	10.6%	*0.08*	2	1.3%	*0.1*
pT2 + unknown		119	32	26.9%		14	11.8%		20	16.8%		2	1.7%	
pT3 + pT4		9	4	44.4%		2	22.2%		3	33.3%		1	11.1%	
**Tumor grade**														
Poor		131	36	27.5%	*0.60*	21	16.0%	*0.06*	21	16.0%	*0.56*	3	2.3%	*0.84*
Unknown		81	20	24.7%		9	11.1%		11	13.6%		1	1.2%	
Moderate/Good		67	14	20.9%		3	4.5%		7	10.4%		1	1.5%	
**Tumor cell content**														
30–49%		31	9	29.0%	*0.82*	6	19.4%	*0.38*	4	12.9%	*0.86*	1	3.2%	*0.81*
50–70%		69	16	23.2%		7	10.1%		11	15.9%		1	1.4%	
> 70%		179	45	25.1%		20	11.2%		24	13.4%		3	1.7%	
**Hormone/ growth factor status (RT-qPCR)**														
*ESR1* negative		0	0			0			0			0		
*ESR1* positive		279	70	25.1%		33	11.8%		39	14.0%		5	1.8%	
*PR* negative		62	16	25.8%	*0.88*	9	14.5%	*0.46*	8	12.9%	*0.78*	2	3.2%	*0.93*
*PR* positive		217	54	24.9%		24	11.1%		31	14.3%		3	1.4%	
*HER2* non-amplified		233	62	26.6%	*0.15*	29	12.4%	*0.30*	34	14.6%	*0.61*	4	1.7%	*0.78*
*HER2* amplified		43	7	16.3%		3	7.0%		5	11.6%		1	2.3%	
*CCDC170* negative		26	4	15.4%	*0.23*	2	7.7%	*0.49*	3	11.5%	*0.70*	0	0.0%	*0.47*
*CCDC170* positive		252	66	26.2%		31	12.3%		36	14.3%		5	2.0%	
**Disease-free interval**														
≤ 1 year disease-free		20	7	35.0%	***0.011***	2	10.0%	*0.08*	4	20.0%	***0.006***	0	0.0%	*0.57*
1–3 years disease-free		71	18	25.4%		10	14.1%		14	19.7%		2	2.8%	
> 3 years disease-free		188	45	23.9%		21	11.2%		21	11.2%		3	1.6%	
**Adjuvant endocrine therapy**														
No		279	66	23.7%		33	11.8%		39	14.0%		5	1.8%	
Yes		0	0			0			0			0		
**Adjuvant chemotherapy**														
No		279	66	23.7%		33	11.8%		39	14.0%		5	1.8%	
Yes		0	0			0			0			0		

*ESR1* estrogen receptor alpha, *CCDC170* coiled-coil domain containing 170, *AKAP12* A-Kinase Anchoring Protein 12 gene, *ESR1-CCDC170* ESR1-CCDC170 gene fusion, *ESR1-AKAP12* ESR1-AKAP12 gene fusion, *pT* primary tumor, *pT1* small primary tumor (tumour is 2 cm across or less), *pT2* tumour more than 2 cm but no more than 5 cm across, *pT3* T3 tumour bigger than 5 cm across, *pT4* tumor with phatological stage, *RT-qPCR* Quantitative Real-Time Polymerase Chain Reaction, *PR* progesterone receptor, *HER2* human epidermal growth factor receptor

Statistically significant differences are indicated in bold

### RNA isolation and RT-qPCR

Total RNA isolation from human breast cancer tissue, breast cancer cell line models and quality control were performed as previously described [20]. Next, cDNA was generated by a cycle at 48 °C for 30 min with RevertAid H-minus (Applied Biosystems, Carlsbad, CA), according to the manufacturer's instructions. The cDNA was then pre-amplified for specific genes as previously described [20]. Briefly, 2 µL of cDNA (0.1 to 1 ng/ µL) was subject to a pre-amplification of 15 cycles using a multiple loci target-specific amplification for *ESR1* fusions with *AKAP12*, *ARMT1* and *CCDC170* and two reference genes, the Epithelian Cell Adhesion Molecule (*EPCAM)* and the Hypoxanthine Phosphoribosyltransferase 1 (*HPRT1*), with TaqMan PreAmp Master Mix (Applied Biosystems), as recommended by the manufacturer. Pre-amplified products were then diluted 12-fold in LoTE buffer (3 mM Tris–HCl/0.2 mM EDTA, pH 8.0) prior to downstream analysis. Next, 5 µL diluted pre-amplified samples were subjected to a TaqMan probe based real-time quantitative PCR (qPCR) for each gene combination, according to the manufacturer’s instructions, in a MX3000P Real-Time PCR System (Agilent, Santa Clara, CA). The average expression of *HPRT1* and the epithelial marker *EPCAM* was used as reference to control RNA quality and calculate the expression levels of target genes, as previously described [20]. Only those samples with a ∆Cq > 25 relative to the two reference genes were used for further evaluation of gene fusions, as previously described [24–26]. Additional file 1 describes the primer sets used in the pre-amplification combination, as well as the Taqman qPCR used to quantify the fusions and reference genes. For *ESR1-CCDC170* fusion transcripts, the variants in which exon 2 of *ESR1* is fused to the coding region (exon 2 to 11) of *CCDC170* were examined (E2-E2, E2-E3, E2-E4, E2-E5, E2–E6, E2–E7, E2–E8, E2–E10 and E2-E11). Samples with a ∆Cq > 25 relative to the reference genes were afterwards validated by MultiNA analysis (Shimadzu Europe, Duisburg, Germany). Only those samples with a MultiNA fusion product of the expected size were considered positive for the fusion transcripts (Additional file 2). The detection of *ESR1-CCDC170* fusion transcripts with RT-qPCR and MultiNA analysis was verified and confirmed in a set of fusion-positive reported breast cancer cell lines (Additional files 3, 4 and 5).

### Statistical analysis

All data were entered in SPSS version 24 (IBM Corp., Armonk, NY, USA) to generate the tables and perform the statistical analyses. For contingency tables, the Pearson Chi-Square Test was used. All *P*-values are 2-sided and *P* < 0.05 was considered statistically significant.

## Results

### Association of *ESR1* with its *CCDC170*, *AKAP12* and *ARMT1* fusion partner

The presence of the *ESR1* fusions with *AKAP12*, *ARMT1* and *CCDC170 (exon 2 to exon 11)* was evaluated in breast cancer tissue samples from 732 breast cancer patients. Fusion transcripts were predominantly detected in the *ESR1* + population, with *CCDC170*, *AKAP12* or *ARMT1* fusion transcripts observed in 27.6%, 4.04% and 1.4% of the ER-positive cases respectively, and seen in 2.3%, 0.8% and 0% of the *ESR1*- cases respectively (*P* < 0.001, Fisher’s exact test two tailed. Table 4 and Additional file 6). In ER-positive tumors, full length *ESR1* and *CCDC170* mRNA levels were strongly correlated (*R*^*2*^ = 0.31, *P* < 0.0001) (Additional file 7A) and transcript levels of both were significantly higher in the group of samples with an *ESR1-CCDC170* fusion transcript when compared to the group without [Student T-Test *P* = 0.0316 and 0.0001, respectively (Additional file 7B).

**Table 4:** Prevalence of *ESR1* fusions in the different analyzed cohorts

			**At least one** ***ESR1-CCDC170*** **(exon 2 to 8) fusion**				***ESR1-CCDC170*** **exon 2**			***ESR1-CCDC170*** **exon 8**			***ESR1_AKAP12***				***ESR1-ARMT1***			
	**Total Count**		**no**	**yes**	**%**	**% of total count**	**no**	**yes**	**%**	**no**	**yes**	**%**	**no**	**yes**	**%**	**% of total count**	**no**	**yes**	**%**	**% of total count**
**All samples studied**	**788**	ESR1 negative	128	3	2.3%	**22.0%**	128	3	2.29%	130	1	0.76%	130	1	0.76%	**2.7%**	131	0	0.00%	**1.1%**
		ESR1 positive	487	170	25.9%		565	92	14.00%	556	101	15.37%	637	20	3.04%		648	9	1.37%	
**1st line Tamoxifen**	**235**	ESR1 negative	0	0	0%	**24.7%**	0	0	0%	0	0	0%	0	0	0%	**2.6%**	0	0	0%	**0.4%**
		ESR1 positive	177	58	24.7%		204	31	13.19%	201	34	14.47%	229	6	2.55%		234	1	0.43%	
**1st line AI**	**87**	ESR1 negative	0	0	0%	**35.6%**	0	0	0%	0	0	0%	0	0	0%	**8.0%**	0	0	0%	**3.4%**
		ESR1 positive	56	31	35.6%		68	19	21.84%	70	17	19.54%	80	7	8.05%		84	3	3.45%	
**1st line endocrine cohort**	**322**	ESR1 negative	0	0	0%	**27.6%**	0	0	0%	0	0	0%	0	0	0%	**4.0%**	0	0	0%	**1.2%**
		ESR1 positive	233	89	27.6%		272	50	15.53%	271	51	15.84%	309	13	4.04%		318	4	1.24%	
**Primary cohort**	**566**	ESR1 negative	113	3	2.6%	**17.8%**	113	3	2.59%	115	1	0.86%	115	1	0.86%	**1.9%**	116	0	0%	**0.7%**
		ESR1 positive	352	98	21.8%		403	47	10.44%	392	58	12.89%	440	10	2.22%		446	4	0.89%	
**Primary LNP cohort**	**192**	ESR1 negative	26	0	0.0%	**15.6%**	26	0	0.00%	26	0	0.00%	26	0	0.00%	**2.6%**	26	0	0%	**0.5%**
		ESR1 positive	136	30	18.1%		152	14	8.43%	148	18	10.84%	161	5	3.01%		165	1	0.60%	
**Primary LNN cohort**	**369**	ESR1 negative	87	3	3.3%	**18.7%**	87	3	3.33%	89	1	1.11%	89	1	1.11%	**1.6%**	90	0	0.0%	**0.8%**
		ESR1 positive	213	66	23.7%		246	33	11.83%	240	39	13.98%	274	5	1.79%		276	3	1.08%	
**Normal breast tissue of breast cancer patients**	**36**	ESR1 negative	0	0	0%	**66.7%**	0	0	0%	0	0	0%	0	0		**0.0%**	0	0	0%	**0.0%**
		ESR1 positive	12	24	66.7%		18	18	50.0%	23	13	36.1%	36	0	0.0%		36	0	0.0%	
**Tissue of breast fibroadenoma's**	**16**	ESR1 negative	0	0	0%	**25.0%**	0	0	0%	0	0	0%	0	0	0%	**0.0%**	0	0	0%	**0.0%**
		ESR1 positive	12	4	20.0%		16	0	0.0%	16	4	20.0%	16	0	0.0%		16	0	0.0%	
**Tissue of breast DCIS**	**13**	ESR1 negative	0	0	0%	**7.7%**	0	0	0%	0	0	0%	0	0	0%	**0.0%**	0	0	0%	**0.0%**
		ESR1 positive	12	1	7.7%		13	0	0.0%	13	0	0.0%	13	0	0.0%		13	0	0.0%	
**Normal breast tissue of healthy women**	**10**	ESR1 negative	0	0	0%	**10.0%**	0	0	0%	0	0	0%	0	0	0%	**0.0%**	0	0	0%	**0.0%**
		ESR1 positive	9	1	10.0%		9	1	10.0%	10	0	0.0%	10	0	0.0%		10	0	0.0%	

*ESR1* estrogen receptor alpha, *CCDC170* coiled-coil domain containing 170, *AKAP12* A-Kinase Anchoring Protein 12 gene, *ARMT1* Acidic Residue Methyltransferase 1, *ESR1-CCDC170* ESR1-CCDC170 gene fusion, *ESR1-AKAP12* ESR1-AKAP12 gene fusion, *ESR1-ARMT1* ESR1-ARMT1 gene fusion, *1st* first line treatment, *LNP* lymph node positive disease, *LNN* lymph node negative disease, *DCIS* Ductal carcinoma in situ

Statistically significant differences are indicated in bold

### Prevalence of *ESR1* fusion genes in normal mammary tissue, benign lesions and carcinoma in situ of the breast

**Fig. 2:**
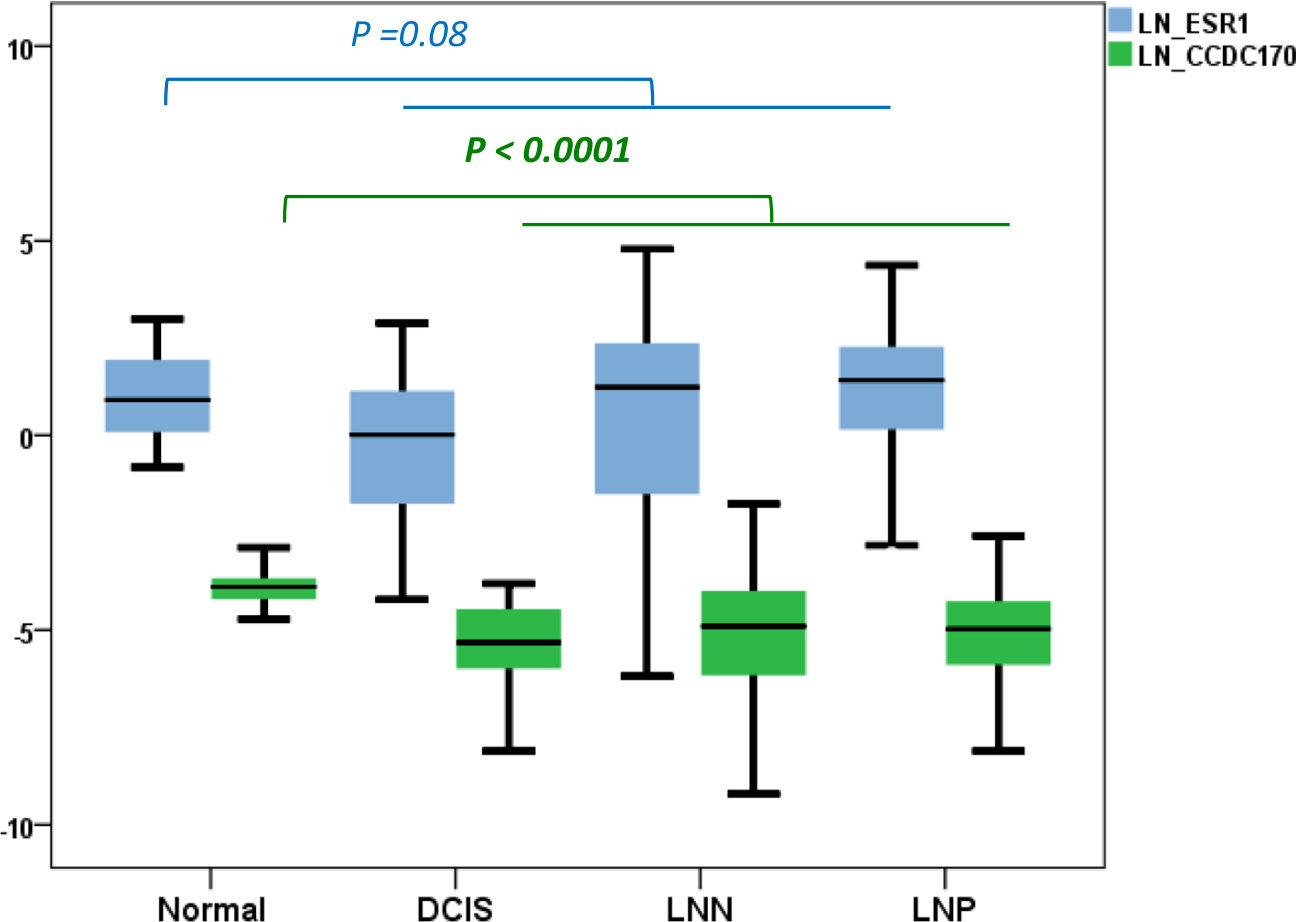
Expression of *ESR1* and *CCDC170* genes in breast tissues. Relative *CCDC170* (blue box) and *ESR1* (green box) mRNA levels normalized to *HPRT1* gene are showed in the y-axis and were measured by RT-qPCR in normal (adjacent to tumor), benign (DCIS) and carcinoma (LNN and LNP) breast tissues. The box plots show interquartile ranges (IQR) together with the median (black horizontal line) of the *ESR1* and *CCDC170* mRNA levels for the different conditions. DCIS: ductal carcinomas in situ; LNP: Lymph node positive; LNN: Lymph node negative

While *AKAP12* and *ARMT1* fusion transcripts were not found in 36 non-malignant breast tissues taken at a distance of the primary tumor, *ESR1*-*CCDC170* fusion transcripts were detected in 67% of these normal breast tissues of patients with diagnosed breast cancer (Table 4). Note that *CCDC170*, but not *ESR1*, mRNA levels were significantly higher in these normal (adjacent to tumor) tissues than in cancer tissue (Kruskal Wallis Test *P* < 0.0001, (Fig. 2). To investigate this unexpectedly high incidence in more detail, we analyzed normal breast tissues of ten women without diagnosed breast cancer, 16 benign fibroadenomas and 13 ductal carcinomas in situ (DCIS) tissues, all of them *ESR1*-positive. In addition, we measured the fusion transcripts in three sets of patient-matched normal breast and primary tumor carcinomas and four patient-matched sets of primary breast tumors and metastatic lymph nodes, also all *ESR1*-positive. In none of these cases did we detect an *ESR1* fusion transcripts with *AKAP12* or *ARMT1*. However, one of the breast tissues of women without breast cancer diagnosis (10%) showed *ESR1*-*CCDC170* exon 2 (E2-E2) fusion transcripts, one of the DCIS cases (7.7%) had *ESR1*-*CCDC170* exon 6 (E2-E6) fusion transcripts, and four patients with fibroadenoma (25%) had ESR1-*CCDC170* exon 8 (E2-E8) fusion transcripts (Table 4 and Additional file 6). For one out of the three matched normal-tumor cases we found an *ESR1*-*CCDC170* exon 8 fusion in both the primary tumor and the normal breast tissue taken at a distance from the primary tumor. Finally, for two out of the four patients of which we had a matched primary tumor and lymph node metastasis, an *ESR1*-*CCDC170* exon 2 fusion was present in both the primary tumor and the lymph node metastasis.

### Prevalence of *ESR1* fusion genes in breast tumor tissues

Since fusion transcripts were predominantly detected in the *ESR1* + population, we decided to investigate the clinical relevance of these transcripts in primary tumors. To this end, we stratified *ESR1* + patients in two distinct cohort: a predictive cohort of advanced BC patients treated with first-line endocrine therapy and a prognostic cohort of primary BC patients with lymph node negative disease (LNN) who did not receive any adjuvant systemic treatment.

In these two *ESR1* + cohorts, *ESR1-ARMT1* fusion transcripts were detected in four patients of the predictive cohort (1.2%) and in three patients of the prognostic cohort (1.1%). Due to the low incidence of this *ESR1-ARMT1* fusion transcript, it was not further pursued. *ESR1*-*AKAP12* fusion transcripts were more common, and observed in 13 patients of the predictive cohort (4.0%) and in five patients of the prognostic cohort (1.8%). The *ESR1*-*CCDC170* fusion transcripts, however, were the most prevalent and detected in the predictive cohort in 89 patients (27.6%) and in the prognostic cohort in 70 patients (25.1%). Interestingly, all patients harboring an *ESR1*-*ARMT1* or an *ESR1*-*AKAP12* fusion were also positive for an *ESR1*-*CCDC170* rearranged transcript*.* Moreover, we noticed the coexistence of the three fusions in two subjects. Of all the breast tissue samples studied, the most prominent *ESR1*-*CCDC170* fusion transcripts found involved exon 2 of *ESR1* fused with exon 2 (14%) and exon 8 (15.37%) of *CCDC170* (Table 4).

### Association of *ESR1* fusion genes with DFS and OS in the prognostic cohort

**Fig. 3:**
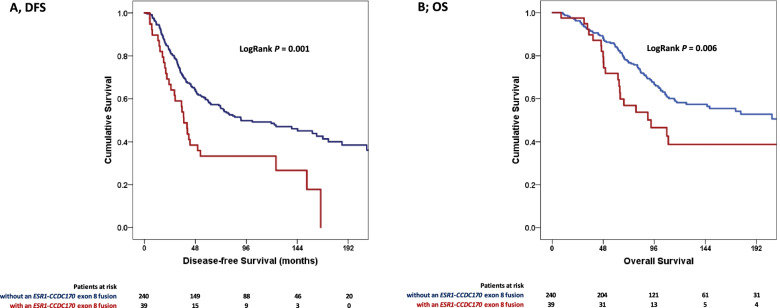
Disease free survival (DFS) and overall survival (OS) in the prognostic cohort. The DFS and OS Kaplan Meier curves in ER-positive LNN patients. **A** DFS of patients with or without *ESR1-CCDC170* exon 8 fusion gene; **B** OS of patients with or without *ESR1-CCDC170* exon 8 fusion gene. The reported *P*-value is from a log-rank test and the test statistics from Cox regression analyses

The presence of *ESR1*-*CCDC170* fusion transcripts in the primary tumor of our *ESR1* + LNN patients predicted a shorter disease-free survival in a Cox proportional hazards regression survival analysis (HR ± 95% CI: 1.44 (1.01 – 2.05), *P* = 0.044) (Table 5). We decided to investigate the two frequently present *ESR1*-*CCDC170* fusion transcripts (E2-E2 and E2-E8). Analyzing the *ESR1*-*CCDC170* exon 2 and exon 8 separately, showed that the fusion with exon 8 of *CCDC170* on its own associated with a short disease free survival (DFS; HR ± 95% CI: 1.95 (1.30 – 2.93), *P* = 0.001). No association with disease free survival was seen for *ESR1*-*AKAP12* fusion transcripts (HR ± 95% CI: 1.23 (0.39 – 3.87), *P* = 0.72). Concerning overall survival, only the presence of an *ESR1*-*CCDC170* exon 8 fusion predicted a shorter overall survival time (HR ± 95% CI: 1.85 (1.18 – 2.90, *P* = 0.007) The DFS and OS Kaplan Meier curves as a function of *ESR1-CCDC170* exon 8 fusion transcripts are shown in Fig. 3A and Fig. 3B, respectively. A multivariate analysis was performed in which age at primary surgery, pathological tumor classification, tumor grade, progesterone receptor and HER2 status were included. The analysis revealed HER2 status as a significant prognostic factor for overall survival, but not for DFS (*P* = 0.36) (Table 5). In this analyses, the presence of *ESR1*-*CCDC170* exon 8 fusion transcripts was an independent prognostic factor for both DFS (HR ± 95% CI: 1.82 (1.20 – 2.75), *P* = 0.005) and OS (HR ± 95% CI: 1.71 (1.08 – 2.72), *P* = 0.001).

**Table 5:** Uni- and multivariate Cox proportional hazards regression survival analysis

		**Univariate model DFS**				**Multivariate model DFS**				**Univariate model OS**				**Multivariate model OS**			
**Parameters**	**n**	**HR**	**(95% CI)**		***P***	**HR**	**(95% CI)**		***P***	**HR**	**(95% CI)**		***P***	**HR**	**(95% CI)**		***P***
	279																
**Age at primary surgery**					*0.25*				*0.31*				*0.19*				*0.18*
≤ 40 years	29	1				1				1				1			
41–50 years	81	0.59	0.35	1.00	***0.049***	0.60	0.35	1.02	*0.06*	0.53	0.30	0.96	***0.036***	0.51	0.28	0.94	***0.032***
51–70 years	125	0.73	0.44	1.19	*0.20*	0.72	0.44	1.18	*0.19*	0.75	0.44	1.28	*0.30*	0.72	0.42	1.26	*0.25*
> 70 years	44	0.78	0.43	1.40	*0.41*	0.71	0.39	1.28	*0.25*	0.73	0.37	1.43	*0.35*	0.73	0.37	1.47	*0.38*
**Menopausal status**																	
Premenopausal	120	1								1							
Postmenopausal	159	1.01	0.73	1.38	*0.96*					1.06	0.74	1.53	*0.73*				
**Pathological Tumor classification**					***0.009***				***0.037***				***0.017***				***0.019***
pT1	151	1				1				1				1			
pT2 + unknown	119	1.54	1.12	2.11	*0.007*	1.35	0.98	1.88	0.069	1.30	0.90	1.87	*0.165*	1.19	0.81	1.74	*0.375*
pT3 + pT4	9	2.31	1.00	5.32	*0.049*	2.47	1.07	5.75	0.035	3.26	1.39	7.62	*0.006*	3.45	1.45	8.19	*0.005*
**Grade**					** < *0.001***				***0.001***				***0.033***				*0.082*
poor	131	1				1				1				1			
unknown	81	1.36	0.97	1.91	*0.076*	1.40	0.98	1.99	*0.064*	0.89	0.59	1.34	*0.577*	0.97	0.64	1.48	*0.894*
moderate and good	67	0.52	0.33	0.82	*0.004*	0.57	0.36	0.89	*0.014*	0.51	0.31	0.85	*0.009*	0.57	0.34	0.94	*0.029*
**ER**	279	1.11	0.98	1.25	*0.10*					0.99	0.86	1.14	*0.92*				
**PR**																	
negative	62	1				1				1				1			
positive	217	0.66	0.46	0.93	***0.019***	0.68	0.47	0.98	***0.037***	0.49	0.33	0.73	** < *0.001***	0.56	0.37	0.85	***0.007***
**HER2 status**^**a**^																	
not amplified	233	1								1				1			
amplified	43	1.21	0.80	1.84	*0.36*					1.82	1.17	2.84	***0.008***	1.72	1.08	2.73	***0.022***
		**Univariate model PFS**								**Univariate model post-relapse survival**							
***1st line Tamoxifen***	235																
at least one *ESR1-CCDC170* (exon 2 to 8) fusion		0.96	0.71	1.30	*0.81*					1.16	0.85	1.60	*0.35*				
*ESR1-AKAP12*		1.37	0.61	3.10	*0.44*					1.92	0.84	4.35	*0.12*				
***1st line AI***	87																
at least one *ESR1-CCDC170* (exon 2 to 8) fusion		0.85	0.53	1.37	*0.50*												
*ESR1-AKAP12*		1.62	0.73	3.60	*0.24*												
						**Separately added to the base model**											
		**Univariate model DFS**				**Multivariate model DFS**				**Univariate model OS**				**Multivariate model OS**			
***at least one ESR1-CCDC170 (exon 2 to 8) fusion***																	
negative	213	1				1				1				1			
positive	66	1.44	1.01	2.05	***0.044***	1.33	0.92	1.92	*0.13*	1.67	1.13	2.47	***0.010***	1.54	1.02	2.33	***0.042***
***ESR1-CCDC170 (exon 2) fusion***																	
negative	246	1								1				1			
positive	33	1.40	0.89	2.21	*0.14*					1.75	1.07	2.87	***0.026***	1.38	0.82	2.33	*0.22*
***ESR1-CCDC170 (exon 8) fusion***																	
negative	240	1				1				1				1			
positive	39	1.95	1.30	2.93	***0.001***	1.82	1.20	2.75	***0.005***	1.85	1.18	2.90	***0.007***	1.71	1.08	2.72	***0.023***
***ESR1-AKAP12***																	
negative	274	1								1							
positive	5	1.23	0.39	3.87	*0.72*					2.45	0.90	6.65	*0.08*				

*DFS* disease free survival, *OS* overall survival, *HR* hazard ratio, *CI* interval of confidence, *pT1* small primary tumor (tumour is 2 cm across or less), *pT2* tumour more than 2 cm but no more than 5 cm across, *pT3* T3 tumour bigger than 5 cm across *pT4* tumor with phatological stage, *ER* estrogen receptor, *PR* progesterone receptor, *HER2* human epidermal growth factor receptor, *AI* aromatase inhibitors, *ESR1-CCDC170* ESR1-CCDC170 gene fusion, *ESR1-AKAP12* ESR1-AKAP12 gene fusion. Statistically significant differences are indicated in bold

^a^ due to unknown data numbers do not add up to 279

### Association of *ESR1* fusion genes with clinical characteristics, PFS and post-relapse overall survival in advanced BC patients

The fusion transcripts were related with traditional clinical parameters, with response to first-line endocrine therapy in the predictive cohort (*n* = 322; tamoxifen (*n* = 235), aromatase inhibitors (*n* = 87)) (Table 2). In the predictive cohort *ESR1-CCDC170* fusion transcripts showed an association with age at start of first-line treatment, whereas *ESR1-AKAP12* fusion transcripts were enriched in patients with progesterone-negative primary tumors at time of surgery and in AI-treated patients who received adjuvant tamoxifen. No relation with PFS after first-line tamoxifen (*n* = 235) was found in our Cox proportional hazards regression survival analysis for the *ESR1*-*CCDC170* fusion transcripts (HR ± 95% CI: 0.96 (0.71 – 1.30), *P* = 0.81) nor for the *ESR1*-*AKAP12* fusion transcripts (HR ± 95% CI: 1.37 (0.61 – 3.10), *P* = 0.44) (Table 5). In addition, the presence of these fusion transcripts did not affect the time from relapse to death (post-relapse survival, HR ± 95% CI: 1.16 (0.85 – 1.60), *P* = 0.35 and 1.92 (0.84 – 4.35), *P* = 0.12, for *ESR1* fusions with *CCDC170* and *AKAP12*, respectively) (Table 5). Similarly, also no association with PFS for first-line aromatase inhibitors (*n* = 87) was found for *ESR1*-*CCDC170* fusion transcripts (HR ± 95% CI: 0.85 (0.53 – 1.37), *P* = 0.50) nor for the *ESR1*-*AKAP12* fusion transcripts (HR ± 95% CI: 1.62 (0.73 – 3.60), *P* = 0.24). With data available for only 27 patients post-relapse, we did not analyze post-relapse survival for aromatase inhibitors. Moreover, no-significant associations with PFS were seen when the *ESR1*-*CCDC170* exon 2 and exon 8 fusion transcripts were analyzed separately (Table 5).

## Discussion

The genetic landscape contributing to de novo or acquired resistance to endocrine therapy in breast cancer patients is not completely understood yet. In this study, we investigated the occurrence of recurrent fusion transcripts between *ESR1* and three different loci adjacent to *ESR1* (*CCDC170*, *AKAP12* and *ARMT1)* and correlated their presence with clinical outcome. All of the fusion transcripts analyzed are recurrent and most frequently present in ER-positive disease and among them *ESR1-CCDC170* fusion transcripts were the most predominant. As proposed by others [10, 13], the presumption was that these fusion transcripts, which are considered to cause constitutive ER signaling, might signify resistance to endocrine therapy. However, in patients with advanced breast cancer, we did not find that the presence of any of these fusion transcripts is associated with outcome to endocrine therapy whether it concerned first line tamoxifen or an aromatase inhibitor. Importantly, smaller size effects from these the variants may be undetected due to the relatively small sample size of the study cohort, 87 patients treated with aromatase inhibitors and 235 subjects with tamoxifen. In contrast, in patients with primary BC and not receiving adjuvant systemic hormone treatments, we found that fusion between *ESR1* and *CCDC170* in general, and between exon 2 of *ESR1* and exon 8 of *CCDC170* in particular, predicted in uni- and multivariable analyses shorter disease free survival as well as shorter overall survival. Thus, *ESR1* and *CCDC170* fusion transcript pinpoint cancers with an adverse outcome.

Understanding the molecular mechanisms that underlay the origin of fusion transcripts could help to comprehend the role of these fusions in carcinogenesis as well as improve the diagnosis of cancer patients [10, 13]. Although the progress in DNA sequencing enhanced detection of recurrent and pathological breast cancer fusions, the complexity of underlying genomic rearrangement patterns makes their characterization at the DNA level often difficult. The fusion between *ESR1* and its neighboring gene *CCDC170* are potentially generated by tandem duplication [9, 13, 27, 28], which is also causing other genetic rearrangements in cancer [9, 29, 30]. Kim et al*.* found a region within the *ESR1* genomic locus most vulnerable to DNA strand breakage, which often included intron 6 region of its neighboring gene CCDC170, resulting in oncogenic mRNA *ESR1*-*CCDC170* fusion transcript of exons 2 of *ESR1* connected to exon 2–11 of *CCDC170,* i.e. the C-terminal domain of CCDC170 [31]. Irrespective of mechanisms causing the gene fusions, they occur in a patient-specific manner, which makes their identification at the DNA level less suitable for routine diagnostics. Our method to analyze fusion transcripts is much less dependent on exact position of the underlying gene fusion at the DNA level and is therefore better suited to evaluate as a general biomarker in large patient cohorts. However, an important caveat for detecting gene fusions at the transcript level is the fact that it cannot distinguish between fusion transcripts arising from actual genetic rearrangements and those that arise from transcription reading from one gene into the next without a genetic cause. Interestingly, Giltnane et al. rejected the option of a run-on transcription for these genes since the 5’end of ESR1 is fused to the 3’ends of *CCDC170* and *AKAP12*, which are upstream of *ESR1* gene [10]. Finally, the generation of artefactual fusion sequences, which are randomly ligated during the sequencing procedure, might happen, as previously reported by *Veeraraghavan *et al. [13]. Overall, we performed RT-qPCR analysis and investigated RNA not DNA, therefore we cannot tell whether fusion transcripts are the results of (DNA) rearrangements. Furthermore, to our great surprise, *ESR1-CCDC170* and *ESR1*-*AKAP12* fusions were detected in ER-negative patients even if at low frequency (2.3% and 0.8%, respectively). Besides sampling bias, this finding might be explained by a challenge in ER and PR determination. Althought immunohistochemistry (IHC) is the “gold standard” to determine the surrogate markers ER and PR for breast cancer classification, several studies addressed limitations in IHC by shedding light on the discordance rates in scoring hormone receptor status with negative and false-positive rates in ER and PR statuses higher than 20% [32, 33]. Similarly, a recently article by *Fakhri et all*. found that 12.5% of samples negative for ER by IHC were positive via microarray analysis [34]. In this context, we performed RT-qPCR to accurately determine hormone receptor status. However, this method could be subject to bias during RNA measurement. Moreover, a recently study found that in primary breast cancers, the ER-negative phenotype is not the result of mutations in ER gene, but is due to deficient ER expression at the transcriptional or post-transcriptional level [35]. Therefore, we might hypothesize that the ER expression might be restored in ER-negative patients due to the strongly impact of the signaling environment, as already demonstrated for breast cancer cells via inhibition of DNA methylation or histone deacetylation [36].

Another interesting question regards the biological significance of clinically relevant fusion transcripts. Gene fusions and their products (RNAs and proteins) are assumed to be exclusive to cancer. However, RNA-sequencing analyses from normal appearing margins of cancerous specimens showed fusion transcripts also in normal tissues [37]. In fact, oncogenic rearrangements, such as the *EML4-ALK* [38], *NPM-ALK* [39], *JAZF1-JJAZ1* [40] and *BCR-ABL1* [41] fusions are also expressed at a low level in histologically non-neoplastic tissues [9]. In our study, expression of *ESR1* fused to exons 2 and exon 8 of *CCDC170* was found in mammary epithelial tissues derived from women without diagnosis of breast cancer, and in cases with (benign) fibroadenomas, respectively. Also in early stages of breast cancer, like DCIS, we detected fusion transcripts. Moreover, *ESR1-CCDC170* fusion transcripts were also detected in normal breast tissues of patients with diagnosed breast cancer. This argues that a percentage may be transcript read-through instead of fusion transcripts arising from gene fusions.

According to our results, the expression of *ESR1-CCDC170* exon 2 and exon 8 fusion transcripts were linked to a less favorable disease in BC patients who not received adjuvant systemic treatment. Overall, our results are in agreement with those reported by *Veeraraghavan *et al. which showed that *ESR1-CCDC170* fusions, when introduced into ER-positive breast cancer cells, leads to a markedly increase of cell motility and colony-forming ability, increase in S-G2/M phase cells and a decrease in G0/G1 phase cells. Although several functional studies [9, 42] demonstrated a role of *ESR1-CCDC170* fusions in endocrine therapy resistance, no relationship between fusion transcripts and treatment outcome was observed in our predictive cohort. Overall, since *ESR1-CCDC170* fusions in our study demonstrated no predictive value for endocrine therapy resistance, their prognostic value might be explained by the recurrent incidence of read-through events during cell cycle progression. This latter has been exemplified with the abundance of *CTSD-IFITM10* readthrough fusions during breast cancer cell proliferation [43].

## Conclusions

The most important conclusion from our work is that among the fusion transcripts evaluated measuring *ESR1-CCDC170* exon 8 fusion transcripts in primary breast cancers has diagnostic potential as it identifies a more aggressive subset of ER-positive breast cancer patients. Furthermore, with our study we demonstrated that *ESR1-CCDC170* fusion transcript does not predict endocrine therapy resistance in our setting.

## Supplementary Information

**Additional file 1.** Assays used for *ESR1-CCDC170* fusion analyses. F: forward primer; R: reverse primer

**Additional file 2.** Expected *ESR1-CCDC170* fusion products. In blue are shown the expected sizes of fusion products while in violet are depicted aspecific products, which can be generated during RT-qPCR

**Additional file 3.** Expression of CCDC170 wildtype and fusion protein evaluated by immunohistochemical staining (IHC) and western blotting in breast cancer cell lines. **A. **IHC performed on a cell line microarray of 44 breast cancer cell lines show a histoscore correlation between the cytoplasmic CCDC170 and CCDC170 wildtype as well as between ESR1-CCDC170 exon 8 fusion transcript levels and CCDC170 wildtype, but not with exon 2 fusion transcript levels. **B.** Western blotting analyses demonstrated the expression of *CCDC170* fusion protein. The exon 2 ESR1 – exon 8 CCDC170 fusion product (~30kD) was detected in ZR75.1 and HCC1500, but not in MCF-7. The exon 2- exon 10 CCDC170 fusion protein (~14kD) was also observed, but only in HCC1500

**Additional file 4.** *ESR1-CCDC170* fusions confirmation by MultiNA in a subset of Breast cancer cell lines. If the Taqman probe-based RT-qPCR generated a positive Cq value, the expected fusion gene product sizes were validated by MultiNA. Only products with a MultiNA fusion product of the expected size and a ∆Cq ≥ -25 relative to the two reference genes were considered positive for the fusion product. MultiNA analyses confirmed the *CCDC170* RNA fusion products in three breast cancer cell lines. Red boxes indicate fusion expression with correct fragment sizes

**Additional file 5.** Expression of *ESR1* fusions and reference (*ESR1* and *CCDC170*) genes in a panel of 54 breast cancer cell lines. Genes with expression level above the threshold are indicated in orange

**Additional file 6.** Details of prevalence of *ESR1-CCDC170* (exon 1-11) fusion genes in the different analyzed cohorts. ESR1: Estrogen Receptor 1 gene; AI: Aromatase Inhibitor; LNP: Lymph node positive; LNN: Lymph node negative; DCIS: ductal carcinomas in situ

**Additional file 7.** *ESR1* and *CCDC170* wildtype expression in ER-positive tumors compared between *CCDC170* fusion-negative and positive cases. **A**. Correlation between *CCDC170* and *ESR1* wildtype expression measured by RT-qPCR. **B. ***CCDC170* and *ESR1* wildtype mRNA levels were measured by RT-qPCR in samples with *ESR1-CCDC170* fusion transcript and compared to the group without fusion transcript. The box plots show interquartile ranges (IQR) together with the median (black horizontal line) of the *ESR1* and *CCDC170* mRNA levels for the different conditions. Group 0: *CCDC170*-fusion negative cases (*n* =387); Group 1: *CCDC*170-fusion positive cases (*n *=159)

## Data Availability

All data generated or analysed during this study are included in this published article and its supplementary information files.

## Abbreviations

AI: Aromatase inhibitor
ARMT1: Acidic Residue Methyltransferase 1
AKAP12: A-Kinase Anchoring Protein 12
BC: Breast cancer
CCDC170: Coiled-Coil Domain Containing 170
DCIS: Ductal carcinomas in situ
DFS: Disease- free survival
ER: Estrogen receptor
ERBB2: Erb-B2 Receptor Tyrosine Kinase 2
ESR1: Estrogen receptor alpha
ET: Endocrine therapy
HPRT1: Hypoxanthine Phosphoribosyltransferase 1
HR: Hazard ratio
IHC: Immunohistochemical staining
LBD: Ligand-binding domain
LNN: Lymph node negative
LNP: Lymph node positive
OS: Overall survival
PFS: Progression free survival
PR: Progesterone receptor
RT-qPCR: Reverse transcriptase quantitative PCR
TAM: Tamoxifen
