# Supplementary figures and images for: The prognostic and predictive value of ESR1 fusion gene transcripts in primary breast cancer
Source: BMC Cancer. 2022 Feb 12;22:165. doi: 10.1186/s12885-022-09265-1 (PMC8840267; doi:10.1186/s12885-022-09265-1)

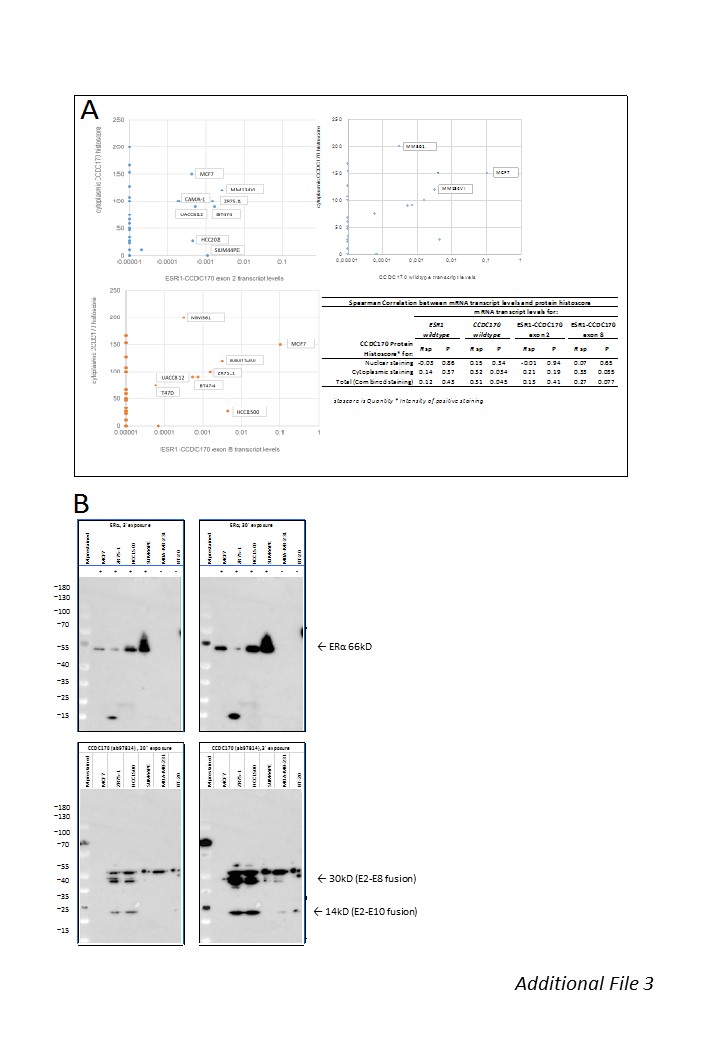

Supplement: Supplementary file 3 — Additional file 3. Expression of CCDC170 wildtype and fusion protein evaluated by immunohistochemical staining (IHC) and western blotting in breast cancer cell lines. A. IHC performed on a cell line microarray of 44 breast cancer cell lines show a histoscore correlation between the cytoplasmic CCDC170 and CCDC170 wildtype as well as between ESR1-CCDC170 exon 8 fusion transcript levels and CCDC170 wildtype, but not with exon 2 fusion transcript levels. B. Western blotting analyses demonstrated the expression of CCDC170 fusion protein. The exon 2 ESR1 – exon 8 CCDC170 fusion product (~30kD) was detected in ZR75.1 and HCC1500, but not in MCF-7. The exon 2- exon 10 CCDC170 fusion protein (~14kD) was also observed, but only in HCC1500 [file 12885_2022_9265_MOESM3_ESM.jpg]

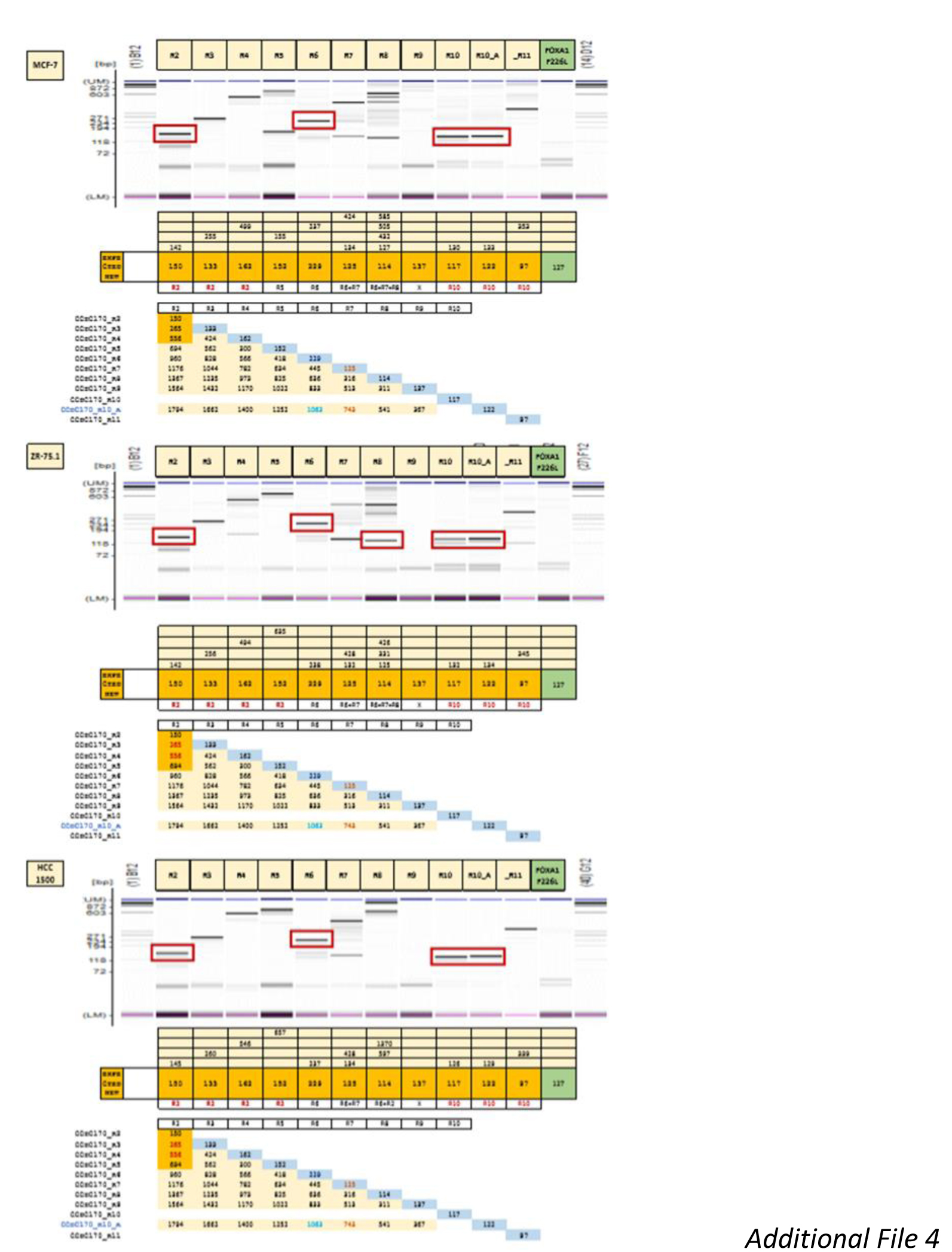

Supplement: Supplementary file 4 — Additional file 4. ESR1-CCDC170 fusions confirmation by MultiNA in a subset of Breast cancer cell lines. If the Taqman probe-based RT-qPCR generated a positive Cq value, the expected fusion gene product sizes were validated by MultiNA. Only products with a MultiNA fusion product of the expected size and a ∆Cq ≥ -25 relative to the two reference genes were considered positive for the fusion product. MultiNA analyses confirmed the CCDC170 RNA fusion products in three breast cancer cell lines. Red boxes indicate fusion expression with correct fragment sizes [file 12885_2022_9265_MOESM4_ESM.jpg]

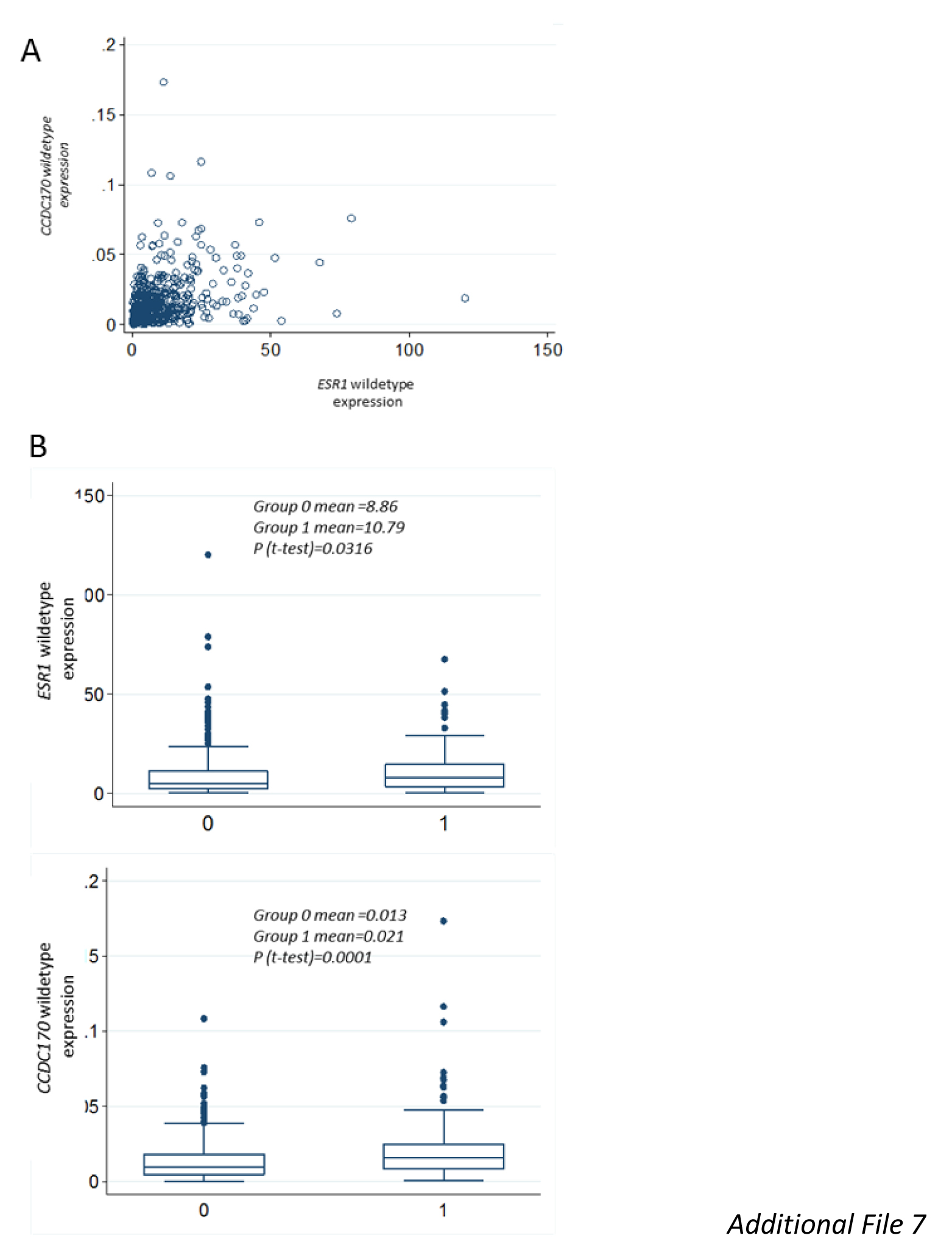

Supplement: Supplementary file 7 — Additional file 7. ESR1 and CCDC170 wildtype expression in ER-positive tumors compared between CCDC170 fusion-negative and positive cases. A. Correlation between CCDC170 and ESR1 wildtype expression measured by RT-qPCR. B. CCDC170 and ESR1 wildtype mRNA levels were measured by RT-qPCR in samples with ESR1-CCDC170 fusion transcript and compared to the group without fusion transcript. The box plots show interquartile ranges (IQR) together with the median (black horizontal line) of the ESR1 and CCDC170 mRNA levels for the different conditions. Group 0: CCDC170-fusion negative cases (n =387); Group 1: CCDC170-fusion positive cases (n =159) [file 12885_2022_9265_MOESM7_ESM.jpg]
